# Revealing *Corynebacterium glutamicum* proteoforms through top-down proteomics

**DOI:** 10.1038/s41598-023-29857-6

**Published:** 2023-02-14

**Authors:** Reynaldo Magalhães Melo, Jaques Miranda Ferreira de Souza, Thomas Christopher Rhys Williams, Wagner Fontes, Marcelo Valle de Sousa, Carlos André Ornelas Ricart, Luis Henrique Ferreira do Vale

**Affiliations:** 1 Laboratory of Protein Chemistry and Biochemistry, Department of Cell Biology, Institute of Biology, University of Brasilia, Brasilia, Brazil; 2 Laboratory of Plant Biochemistry, Department of Botany, Institute of Biology, University of Brasilia, Brasilia, Brazil

**Keywords:** Biochemistry, Biotechnology, Cell biology, Microbiology

## Abstract

*Corynebacterium glutamicum* is a bacterium widely employed in the industrial production of amino acids as well as a broad range of other biotechnological products. The present study describes the characterization of *C. glutamicum* proteoforms, and their post-translational modifications (PTMs) employing top-down proteomics. Despite previous evidence of PTMs having roles in the regulation of *C. glutamicum* metabolism, this is the first top-down proteome analysis of this organism. We identified 1125 proteoforms from 273 proteins, with 60% of proteins presenting at least one mass shift, suggesting the presence of PTMs, including several acetylated, oxidized and formylated proteoforms. Furthermore, proteins relevant to amino acid production, protein secretion, and oxidative stress were identified with mass shifts suggesting the presence of uncharacterized PTMs and proteoforms that may affect biotechnologically relevant processes in this industrial workhorse. For instance, the membrane proteins mepB and SecG were identified as a cleaved and a formylated proteoform, respectively. While in the central metabolism, OdhI was identified as two proteoforms with potential biological relevance: a cleaved proteoform and a proteoform with PTMs corresponding to a 70 Da mass shift.

## Introduction

The bacterium *Corynebacterium glutamicum* is an industrial workhorse capable of producing a broad range of biomolecules from a large pool of substrates^1^ and is currently the best option for production of L-alpha amino acids^2^. Amino acid production represents a multi-billion dollar market^3^, with an annual production of approximately 10 million tons worldwide^2^, with l-glutamate and l-lysine corresponding to 3.3 million tons/year and 2.2 million tons/year, respectively^2^. Besides amino acid production, *C. glutamicum* is also utilized in other biotechnological processes, such as the production of heterologous proteins^4^, organic acids^5^, carotenoids, isobutanol^6^, polymers^7^, and more recently, its potential in bioremediation processes has been investigated^8^.

Recent developments in proteomics have demonstrated the importance of post-translational modifications (PTMs) in several species of bacteria^9^. In *C. glutamicum*, the importance of PTMs was investigated previously revealing the crucial effect of oxoglutarate dehydrogenase inhibitor (OdhI) phosphorylation in the production of l-glutamate^10,11^. More recently, mass spectrometry proteomics of *C. glutamicum* showed the influence of l-glutamate-producing conditions on the succinylation and acetylation of *C. glutamicum* metabolic proteins, including OdhI^12^.

There are currently two main proteomics approaches that are referred to as bottom-up and top-down. Briefly, in the bottom-up approach proteins are cleaved by a protease (usually trypsin) and the subsequent peptides are analyzed by mass spectrometry, whilst in the top-down procedure, proteins are examined in their intact forms^13^. As a consequence of protease digestion, the information from several protein regions can be lost in bottom-up approaches, including PTMs. Furthermore, in this approach protein identity is inferred based on peptide identification, and this may cause ambiguity in cases in which peptides may belong to more than one protein^13^. In contrast, the top-down approach allows the identification of the full sequence of proteins. Moreover, analysis of intact forms of proteins permits the identification of proteoforms, defined as different forms of proteins derived from the same gene^14^. Such proteoforms can be generated by amino acid residue substitution, proteolytic cleavage, alternative splicing, and several types of PTMs^15^. Furthermore, different proteoforms of the same protein can have different function and affect biological processes of an organism^16^. The power of top-down proteomics has been applied to other bacteria^17^. For instance, top-down proteomics of *Escherichia coli* and the identification of proteoforms has been performed^18^. Despite the evidence of the relevance of PTMs in *C. glutamicum* metabolism, no top-down proteomic studies have been published to date. Here we carried out the first top-down proteomics analysis of *C. glutamicum*, using a precursor tolerant approach to identify and reveal PTMs and proteoforms.

## Results and discussion

**Figure 1:**
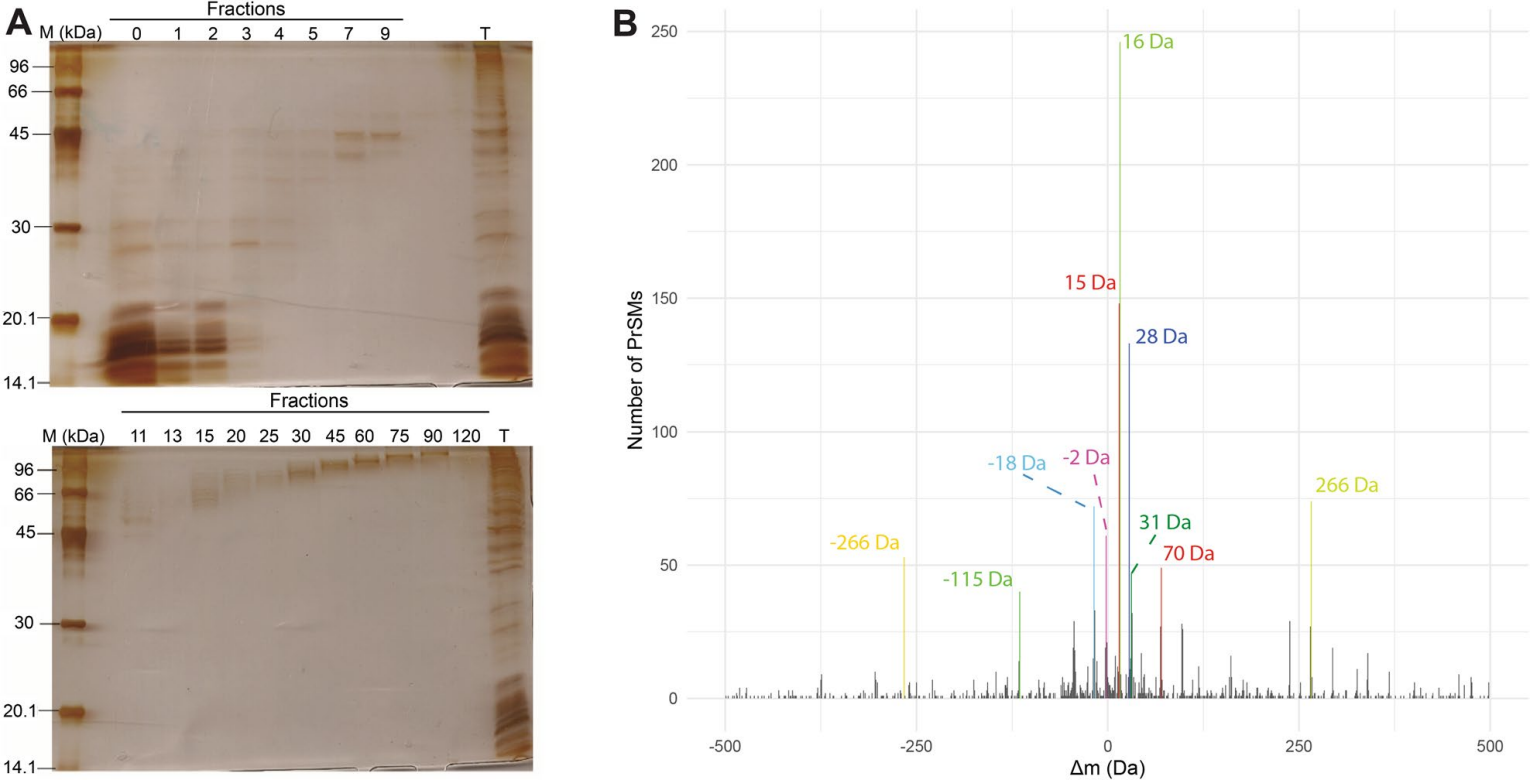
SDS-PAGE of GELFrEE fractions and global profile of identified mass shifts (Δm). (**A**) SDS-PAGE of fractions resulting from GELFrEE (represented by the numbers) and pre-fractionated sample (T). (**B**) Difference of observed and theoretical precursor masses (mass shifts, Δm) PrSMs count histogram, where the ten most frequent Δm are highlighted in different colors. The bin was set to 1 and x limits to −500 Da and 500 Da.

As expected, a wide range of proteoform molecular masses could be seen in the pre-fractionated intracellular proteins, but GELFrEE was efficient in separating large proteoforms from smaller forms (Fig. 1A). Fractions 0, 1, 2, 3, 4, 5, 7, 9 and 11 displayed proteoforms below 50 kDa, and for this reason were chosen for subsequent LC–MS/MS analysis. Despite the clear presence of proteins in the 30–50 kDa range, as shown by SDS-PAGE (Fig. 1A), LC–MS analysis detected proteoforms predominantly below 30 kDa (supplementary Fig. S1). The reduced number of observed proteoforms larger than 30 kDa may be due to known challenges for the identification of denatured large proteoforms such as the signal-to-noise ratio reduction occurring with increases in proteoform molecular weight^19^.

### Global post-translational modifications profile

The top-down proteomic analysis of *C. glutamicum* generated 5127 proteoform spectrum matches (PrSMs), providing the identification of 1125 different proteoforms related to 273 different proteins (supplementary Table 1). Approximately 65% (177 proteins) of the total protein number and 47% of PrSMs (2423 PrSMs) were identified with mass shifts (Δm), mass differences between the expected precursor mass and observed precursor mass, which suggests the presence of PTMs. Putative PTMs were annotated in supplementary Table 1 using the entire Unimod database, however, this information should be used with caution, since spectra were not manually verified and the software considered rounded mass shifts for comparisons, generating a tolerance of approximately 0.5 Da. There are software available for spectra visualization and further evaluation of PTMs, which includes ProSight Lite^20^ and TopMSV^21^. Analysis of all PrSMs with Δm revealed a broad diversity of Δm, suggesting the presence of different PTMs (Fig. 1B).

Some of the most frequent Δm identified here were also reported as highly present in *Escherichia coli* through bottom-up proteomics using an unbiased identification strategy^22^. In *C. glutamicum,* the mass shifts most frequently identified were 16 Da (putative oxidation, 10.15% of modified PrSMs), −18 Da (putative dehydration, 2.97% of modified PrSMs), and 32 Da (possible double oxidation, 1.32% of modified PrSMs). Interestingly, the proportion of *E. coli* identified peptide spectrum matches (PSMs) with -18 Da (3.8%) and 32 Da (0.8%) Δm were very similar to those found in this study. In contrast, the proportion of PSMs identified with 16 Da (24%) Δm in *E. coli* was greater than in *C. glutamicum.* However, it is worth mentioning that the high number of PrSMs identified with 15 Da Δm (6.11% of modified PrSMs) may be caused by miss identification of the 16 Da Δm, as will be further discussed in the next topics.

Proteins identified with Δm were analyzed using the String Cytoscape app, after clustering proteins according to their interaction nodes, each cluster was submitted to overrepresentation analysis and the most representative terms were related to different gene ontology terms such as protein-containing complexes, electron transfer activity and CYTH domain (CyaB, thiamine triphosphatase), pyrimidine metabolism, transmembrane helix, biosynthesis of secondary metabolites and thioredoxin domain. Moreover, eight ribosomal PrSMs presented two Δm, indicating the presence of at least two concomitantly occurring PTMs (supplementary Fig. S2).

**Figure 2:**
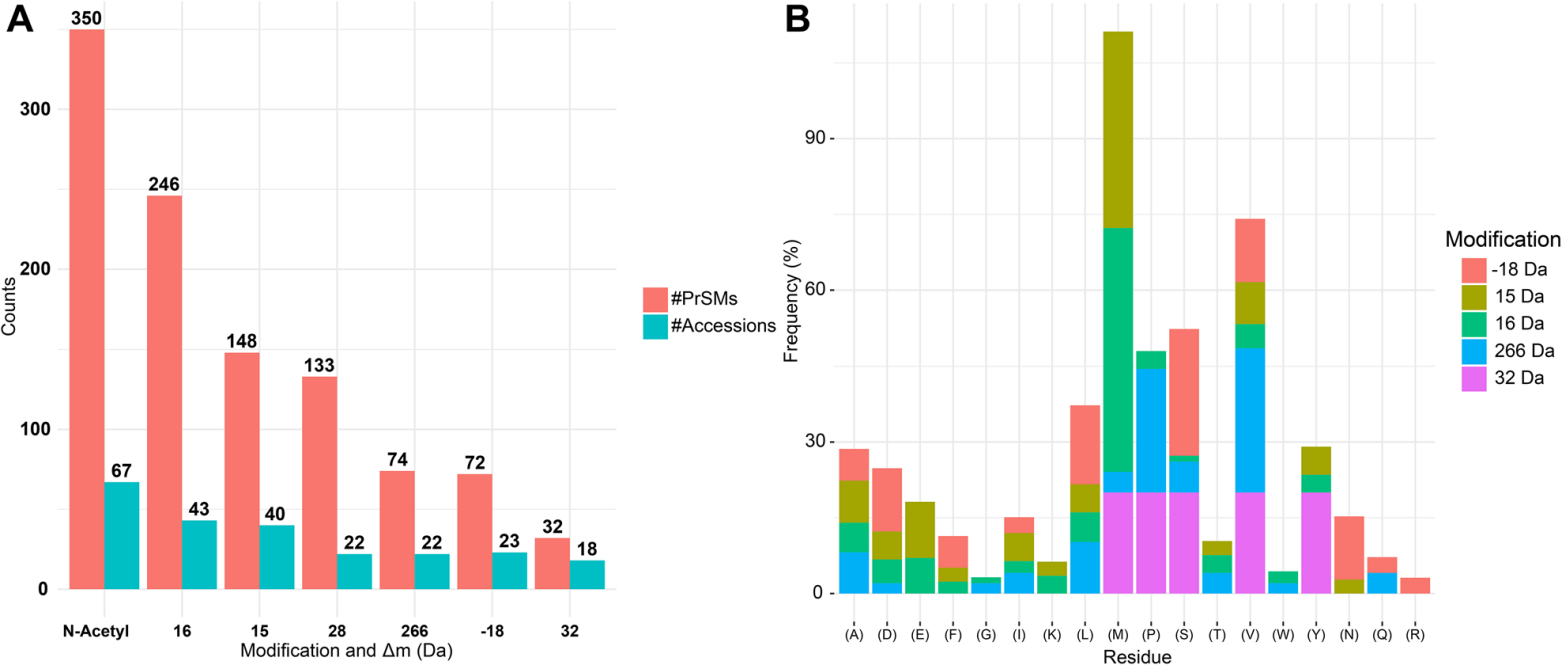
Number of identified PrSMs and protein accessions of the most frequent modifications and ratio of unambiguously identified residues of each mass shift. (**A**) Number of proteoform spectrum matches (PrSMs) and proteins identified with most frequent mass shifts and N-terminal acetylation. (**B**) Ratio of the number PrSMs of an amino acid residue modified by each mass shifts (Δm) and the total PrSMs identified with the same Δm. For example, the number of methionine identified with mass shift of 16 Da (41 PrSMs)/number of all residues identified with 16 Da Δm (85 PrSMs) = 48% (represented by the light green bar above the (M) in the x axis).

Beyond functional annotation, we analyzed the number of N-terminal acetylation and some frequently identified Δm: 16 Da, 15 Da, 28 Da, 266 Da, -18 Da, and 32 Da (Fig. 2A). These Δm suggest the presence of oxidation (UnimodAC: 35, Δm = 15.994915 Da), deamidation followed by a methylation (UnimodAC: 528, Δm = 14.999666 Da), formylation (UnimodAC: 122, Δm = 27.994915 Da), sodium dodecyl sulfate (SDS) adducts^23^, dehydration (UnimodAC: 23, Δm = −18.010565 Da), and persulfide (UnimodAC: 421, Δm = 31.972071 Da). The 28 Da mass shift may also correspond to di-methylation (Unimod: 36, Δm = 28.031300 Da). Despite being difficult to differentiate between formylation and di-methylation, the higher number of Δm close to 27.99 Da and 27.98 Da (supplementary Table 2) leans the inference towards formylation events. The amino acid residue localization of the 28 Da mass shift was not possible without ambiguity, as can be seen by the absence of this mass shift in Fig. 2B. A possible reason for this is that the suggested formylation events were identified mainly at the N-terminal of proteins, and its assignment was always between a few N-terminal residues of the protein, as in the case of the protein MscL (supplementary Fig. 7). When the assignment of the mass shift was in more than one amino acid residue, the information was not plotted to avoid ambiguity. The suggested oxidation events related to the 16 Da Δm is further supported by a large number of methionine residues modified by this mass shift (Fig. 2B). On the other hand, the 15 Da Δm seems to be sometimes misinterpreted, since the most frequently identified residue with this modification was also methionine. However, some glutamic acid residues were identified with this Δm, suggesting that deamidation followed by methylation can still be present in some cases, but are easily confused with oxidations (Fig. 2B). The 32 Da mass shift identified in some proteins could be related to persulfide PTM, however, this modification occurs in cysteine and aspartic acid, and none of these could be observed in the unambiguously identified residues (Fig. 2B). Other possibility would be two oxidations, supported by the methionine residues identified with this mass shift. On the other hand, the possibility of persulfide modification should not be excluded, mainly in proteins in which the modified amino acid residue could not be unambiguously identified. Protein oxidation may have great relevance in biological processes, however, it is important to note that it may also be an artefact caused by sample handling^24^. Moreover, several Δm were approximately 266 Da, suggesting the presence of adducts resulting from sodium dodecyl sulfate (SDS). The presence of SDS artifacts was also reported in an intact proteomic analysis of *E. coli*, and suggested to be caused by incomplete removal of the detergent^25^. Regarding the −18 Da Δm, it may result from dehydration events and was predominantly identified in serine residues (Fig. 2B). These predominant mass shifts may also be related to other putative PTMs, such as tyrosine oxidation to 2-aminotyrosine (UnimodAC: 342, Δm = 15.010899 Da) for the mass shifts of 15 Da. These possibilities can be observed separately for each identified PrSM in supplementary Table 1. N-terminal acetylation was the most frequently identified PTM (Fig. 2A); on the other hand, the 42 Da mass shift, which suggests lysine acetylation (UnimodAC: 1, Δm = 42.010565 Da) was not frequently detected (supplementary Table 2). In identifying proteoforms, the N-terminal acetylation was used as a variable modification. Therefore, proteoforms with lysine acetylation near the N-terminal without fragments to explain the 42 mass shift to a lysine residue could be misidentified with a N-terminal acetylation.

**Figure 3:**
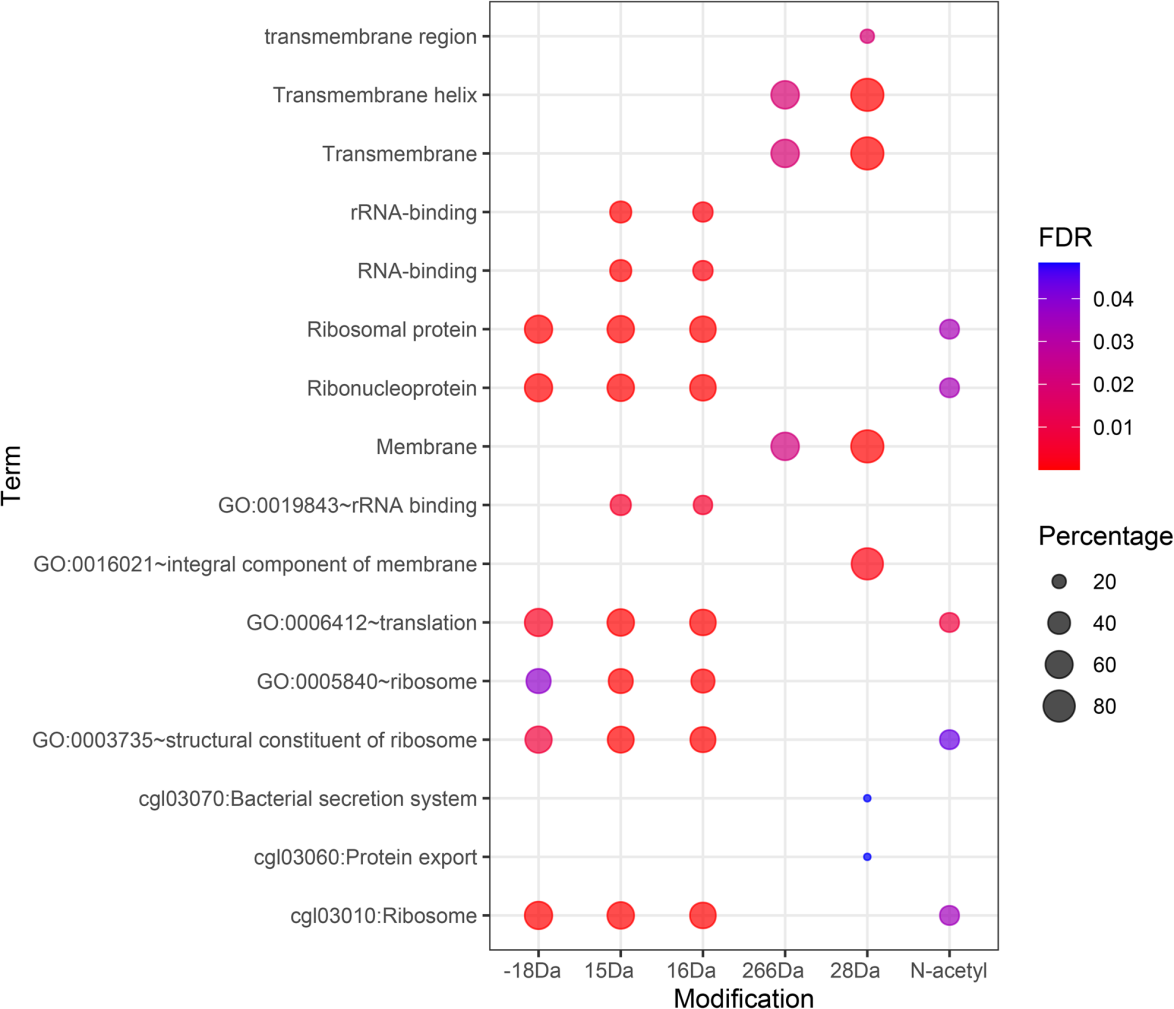
Overrepresentation analysis of proteins according to identified modification and mass shifts. The proteins accession codes of each modification or mass shift were separately submitted to overrepresentation analysis on DAVID against all encoded proteins of the *C. glutamicum* genome. The resulting FDR, representing the score of enrichment, and percentage, representing the proportion of identified proteins to all proteins present in the related *gene-set,* were plotted using ggplot2 bubble plot representation.

Overrepresentation analysis of proteins belonging to the six most frequent modifications allowed the identification of terms related to ribosomal proteins, predominant in proteins with Δm of −18 Da, 15 Da, 16 Da, and N-terminal acetylated proteins (Fig. 3). Prokaryotic ribosomal proteins are described to be commonly methylated and acetylated^26^. On the other hand, Δm of 28 Da and 266 Da were overrepresented for terms related to membrane proteins. In addition, proteins identified with 28 Da mass shift showed overrepresentation for the secretion system and protein export (Fig. 3).

We identified 13 proteins that presented more than 15 proteoforms and most of them were ribosomal (supplementary Fig. S3). Moreover, when considering proteins with more than 9 proteoforms, the cellular component annotation also revealed membrane proteins (supplementary Fig. S3). In despite of the great number of proteoforms, it is worth mentioning that some of them may be boosted by SDS adducts. For example, 50S ribosomal protein L7/L12 (Q8NT28) proteoforms were identified with Δm of 266 Da, 532 Da, and 401 Da, probably representing the addition of one SDS adduct, two SDS adducts, and two SDS adducts plus the loss of the initiator methionine residue. The presence of SDS adducts in proteins is not desirable, however, the identification of these proteins is important to avoid misidentifications and to assign proteins that were only identifiable through adducted forms^25^.

In order to contribute with more accurate data regarding *C. glutamicum* biotechnological and metabolic processes, some PrSMs related or potentially involved with these functions were manually characterized through inspection of MS1 and MS2 Spectra (Table 1).

**Table 1:** Proteoforms with potential metabolism regulation function and of biotechnological interest.

UniprotAC	Protein name	Biological Process	Proteoforms	Putative PTM
Q8Z469	SecG	Protein export	28 Da	N-formylation
Q8NS24	mepB	Peptidase/cell wall metabolism	N-terminal cleavage	N-terminal cleavage
Q8NQJ3	OdhI	Glutamate production	70 Da/N-terminal cleavage	Crotonaldehyde lipid peroxidation or butyrylation
Q8NL68	HMADP	Glutamate production/stress response	30 Da/−2 Da	Methylation + oxidation/disulfide bond
Q8NMS6	Peroxiredoxin	Stress response	154 Da/186 Da	ONE lipid peroxidation/ONE lipid peroxidation + oxidation
Q8NLG6	Thioredoxin	Stress response	30 Da/-2 Da	Methylation + oxidation/disulfide bond
Q8NS07	MscL	Metabolite efflux	28 Da	N-formylation

Proteoforms that presented a potential involvement in the regulation of metabolism or important biotechnological processes are represented by their corresponding protein names, Uniprot accession code (AC) and identified mass shift or sequence cleavage. Putative post-translational modifications (PTM) were defined based on identified mass shifts using the unimod database (https://www.unimod.org).

### Membrane proteins

**Figure 4:**
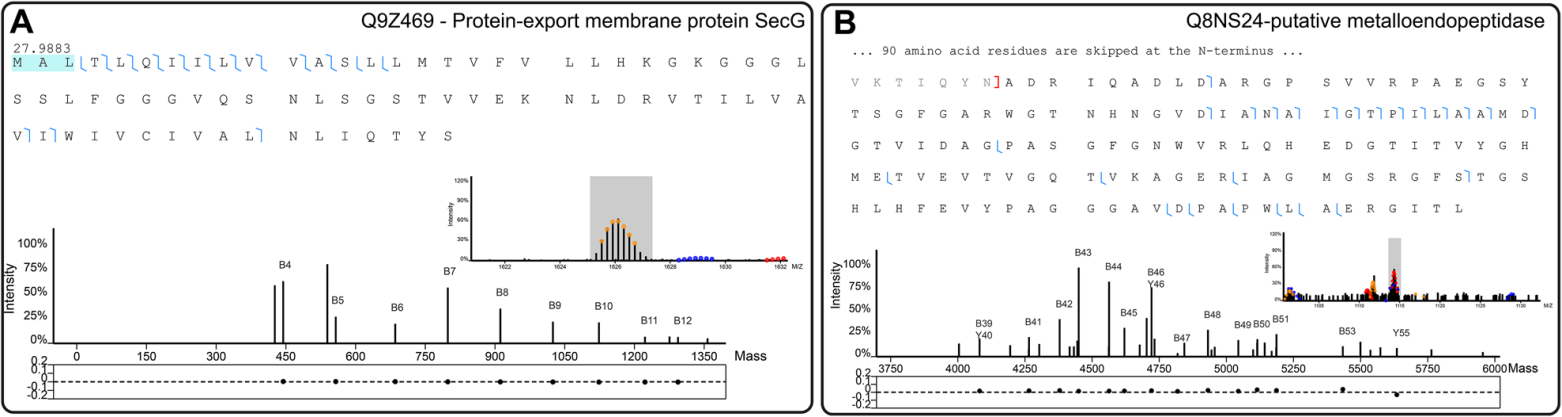
PrSMs of Protein-export membrane protein SecG (Q9Z469) and putative metalloendopeptidase mepB (Q8NS24). In the top region of both images (A and B) can be seen the sequence of the identified protein, where possible sites of the Δm are represented by residues highlighted in blue background and matched fragments are represented by blue lines between amino acid residues. In the bottom region, the MS1 and isolation window of the identified precursor are depicted (right side of each figure) as well as the mass representation of the MS2 spectrum attached to the mass error of matched fragments. (**A**) Inspection of the 28 Da mass shift identified in SecG, where we could see that 12 fragments (all *b*-ions) support the identification of the 27.9883 mass shift near the N-terminal. The mass error of great part of these fragments were close to 0 ppm, as depicted in the bottom of the image. (**B**) Inspection of mepB cleaved proteoform (cleavage site represented by the red bracket), which demonstrates a great number of fragments supporting the cleaved proteoform (all *b*-ions) with mass error close to 0 ppm.

Secretion system protein (SecG) (Fig. 4A) and large-conductance mechanosensitive channel (MscL) (supplementary Fig. S4) were identified with 28 Da mass shifts. The PrSMs fragments of these proteoforms presented ions supporting the identification of a mass shift of 28 Da, always near the N-terminus, and the mass errors of fragments containing this Δm were extremely low. An example of MS1 and MS2 inspection is demonstrated with SecG (Q8Z469) (Fig. 4A). In this figure, the large number of *b-*ions supporting the 27.9883 mass shift (*b4, b5, b6, b7, b8, b9, b10, b11 and b12*) represented by the blue lines in the protein sequence is shown, and it can be seen that the mass shift is localized to a very narrowed region of three amino acid residues, due to the several *b-ions* contained in this region.

The 28 Da mass shift near the protein N-terminus of these proteins suggests the presence of N-terminal formylation (Unimod: 122, Δm = 27.994915 Da) in the identified proteoforms. Recent evidence has suggested the N-terminal formylation of methionine as a signal for protein degradation. This mechanism has been hypothesized as a quality control of protein translation in bacteria^27^. Both the SecG and MscL formylated PrSMs mentioned above were identified with the presence of N-terminal methionine. This suggests a possible mechanism of degradation of membrane proteins, including some with promising biotechnological applications. For example, the SecG protein is part of the Sec protein export pathway. There is a growing interest in the capacity of *C. glutamicum* to express and secrete heterologous proteins of biotechnological interest^4,28^. Furthermore, MscL and MscS are mechanosensitive channels, known for reacting to osmotic stress. Another mechanosensitive channel of *C. glutamicum* (MscCG; P42531) plays a major role in l-glutamate efflux^29^.

Top-down proteomics is efficient in identifying cleaved proteoforms. For example, mepB, a membrane protein related to the metalloendopeptidase (Q8NS24) was identified by a portion of its sequence with precursor mass of 14.464 kDa. This mass corresponds to the loss of 97 amino acid residues from its N-terminal region (Fig. 4B). In contrast, a mepB cleavage site was supposed to be between Ala43 and Ala44, after a putative signal peptide or transmembrane helix^30^. Furthermore, according to pfam (https://pfam.xfam.org/), mepB has a domain belonging to the M23 metallopeptidase family (MEROPS) [130–226]. A well characterized member of MEROPS is the LytM protein from *Staphylococcus aureus*, a metallopeptidase involved in autolysis^31^. It was demonstrated that cleavage in the N-terminal chain causes its peptidase activity to be activated^32^. Moreover, MEROPS proteins have specificity to peptidoglycan polyglycine regions, some with suggested cell wall metabolism activity^33^. In agreement, the *C. glutamicum mepB* gene was described as part of the MtrAB regulon, a two component system implicated in osmoregulation and cell wall metabolism control^34^. Although it is not clear whether mepB is secreted or membrane bound, it has been suggested that its activity would occur extracitoplasmically^30^. Considering this, *C. glutamicum* mepB may have an important role in cell wall metabolism and/or heterologous protein secretion integrity, and its activity is likely regulated by cleavage of its N-terminal.

### Tricarboxylic acid cycle and glutamate metabolism

**Figure 5:**
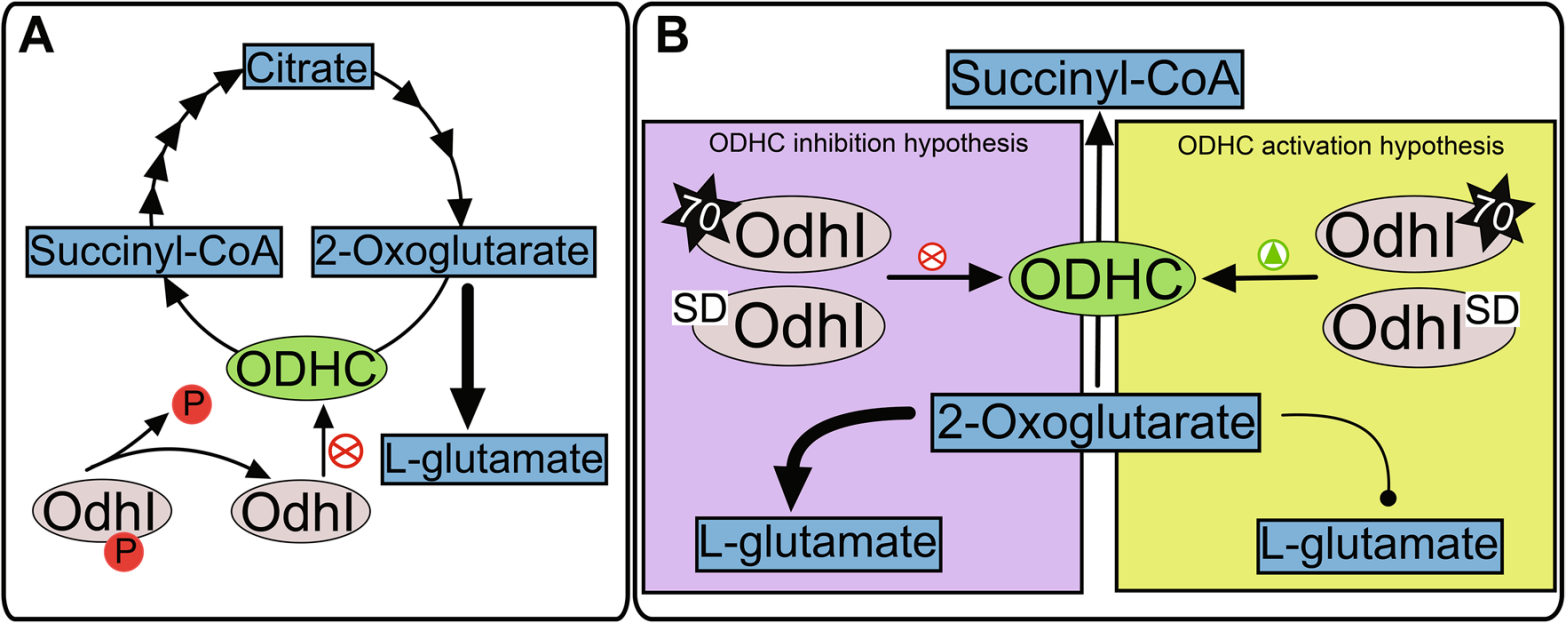
Representation of known and putative OdhI interactions with ODHC and its impact on l-glutamate production. (**A**) Interaction of OdhI and ODHC hampered by OdhI phosphorylation. The absence of the phosphate group on OdhI results in its interaction and inhibition of ODHC (represented by the red symbol), and flux to l-glutamate production is enhanced (represented by the large arrow from 2-oxoglutarate to l-glutamate)^11,37^. (**B**) Depiction of possible metabolic effects and protein interactions affected by OdhI proteoforms described in this study: Δm of 70 Da (represented by the star symbol), and N-terminal dipeptide cleavage (represented by the SD letters on OdhI). The effect of identified proteoforms on OdhI function remains unclear, however potential affected mechanisms are represented through the inhibition of ODHC (purple box; enhancement of L-glutamate production symbolized by the large arrow), or activation of ODHC (green box; decrease of L-glutamate production symbolized by the line with a dote). Both hypothesis may influence l-glutamate production. *OdhI* oxoglutarate dehydrogenase inhibitor, *ODHC* oxoglutarate dehydrogenase complex.

*C. glutamicum* is widely used in the industrial production of amino acids, especially l-glutamate, which is produced in millions of tons per year^35^. The tricarboxylic acid (TCA) cycle is an important step in l-glutamate production by *C. glutamicum*. It is well established that the decrease of 2-oxoglutarate dehydrogenase complex (ODHC) activity occurs in conditions that induce l-glutamate production^36,37^. Furthermore, ODHC activity was found to be regulated by the phosphorylation status of oxoglutarate dehydrogenase inhibitor (OdhI, Q8NQJ3). Thus, the phosphorylated proteoform of OdhI is unable to interact with ODHC, whilst, unphosphorylated OdhI interacts with ODHC inhibiting 2-oxoglutarate conversion to succinyl-CoA^11^ (Fig. 5A). Moreover, the decreased ODHC activity causes an enhanced production of l-glutamate from 2-oxoglutarate^37^ (Fig. 5A). Congruently, OdhI phosphorylation status is affected by methods that induce l-glutamate production^11^*.*

In this study, seven proteoforms of OdhI were identified. Some of them seemed to be caused by sample preparation or ionization process, such as oxidation (Δm = 16 Da) and dehydration (Δm = −18 Da). Conversely, two proteoforms were identified with potential biological relevance: one of them with N-terminal cleavage of three residues and another one with Δm of 70 Da (supplementary Fig. S5). Although these two proteoforms were confidently identified by TopPic, a low number of matching fragments covered the modified regions, lowering our confidence regarding their identifications, principally concerning their location in the sequence. On the other hand, in agreement with one of the identified proteoforms, a bottom-up proteomic analysis in a preliminary study conducted by our group suggested the presence of Δm of 70 Da in the N-terminal peptide of OdhI (data not shown).

Recently, the 70 Da mass shift was identified in the GM-CSF heterologous expressed protein in *Escherichia coli* system. This modification was hypothesized to be a result of crotonaldehyde formed during oxidative stress by lipid peroxidation. The aldehyde reacts with the protein N-terminus or lysine residues, resulting in the Δm of 70 Da^38^. Another possible PTM for this mass shift is the butyrylation of lysine (UnimodAC: 1289, Δm = 70.041865 Da). In the present study, the exact site of the modification resulting in the 70 Da mass shift in OdhI could not be identified due to the complexity of the spectra and limited fragmentation by MS/MS. Therefore, other modifications such as 5 methyl group additions (14 Da), and acetylation (42 Da) followed by formylation (28 Da) could not be excluded. As aforementioned, ODHC inactivation arises from the interaction of unphosphorylated OdhI and the OdhA subunit of ODHC. The T14 OdhI phosphorylation inhibits this interaction, resulting in ODHC activation^10^. OdhI has a fork-head associated (FHA) domain responsible for the recognition of a phosphothreonine residue. Furthermore, this domain is the region that interacts with the OdhA subunit of ODHC, resulting in its inactivation. Moreover, it was demonstrated that the phosphorylation of the OdhI threonine residue causes drastic changes in its conformation, leading to an auto inhibition of its function, consequently activating ODHC^39^. More recently, it was reported that K142 succinylation also affects the OdhI–ODHC interaction, hampering the inhibition of ODHC with impacts on *C. glutamicum* l-glutamate production^12,40^.

Furthermore, as an acetylation site at K52 of OdhI was also described^12,40^, we investigated such a modification in addition to N-terminal formylation (27.9949 Da + 42.0106 Da) as a possible cause of production of the 70 Da OdhI proteoform. However, these modifications resulted in a considerable loss of matched fragment peaks (data not shown). Therefore, it is improbable that this was the source of such a mass shift. Moreover, the suggested region for the 70 Da Δm is near to the known T14 phosphorylation site of OdhI, consequently raising the question if it could affect its interaction with ODHC. OdhI phosphorylation at T14 is mainly catalyzed by *C. glutamicum* PknG^10^. Considering the recognition capacity of the OdhI FHA domain of its phosphothreonine and the close location of this phosphorylation site with the 70 Da Δm proteoform, this modification may affect OdhI inhibition of ODHC, resulting in its activation or inactivation (Fig. 5B). A possible mechanism is the obstruction of OdhI phosphorylation by the presence of the 70 Da PTM, allowing the FHA domain interaction and consequently inhibition of ODHC (Fig. 5B, purple box). Another hypothesis is that the 70 Da modification of OdhI, even having T14 phosphorylated residue, interferes with the FHA domain recognition of the phosphothreonine, resulting in the inhibition ODHC by OdhI (Fig. 5B, purple box). Moreover, the 70 Da mass shift may even behave similarly to the phosphorylated residue, inducing the conformational change of OdhI, inhibiting it and activating ODHC (Fig. 5B, green box). It is less likely that the N-terminal tripeptide truncation causes similar effects, however its influence in OdhI cannot be discarded (Fig. 5B). Furthermore, these alterations may influence l-glutamate production in these bacteria, as a consequence of ODHC regulation (Fig. 5B). Despite these potentially relevant effects of OdhI putative proteoforms, further studies must be performed to investigate the importance and validity of these modifications in OdhI function.

Another protein with relevance to the glutamate production process is the heavy-metal-associated domain (HMA) containing protein (Q8NL68, HMADP). Its corresponding transcript was identified as up-regulated in a series of glutamate overproduction conditions, however its function in this process remains unclear^41^. Here we identified three different proteoforms of this protein. One of them presented Δm of −2 Da (supplementary Fig. S6), suggesting the presence of a disulfide bond (UnimodAC: 2020, Δm = −2.015650 Da), amino acid residue substitution (UnimodAC: 1217, Δm = −2.015650 Da, Val- > Pro or UnimodAC: 1145, Δm = −1.997892 Da, Met- > Glu) or didehydro (UnimodAC: 401, Δm = −2.015650 Da) in the modified region. Moreover, several PrSMs were identified with Δm of approx. 30 Da. Inspection of its spectra resulted in the confirmation of this mass shift, but it is more likely that it resulted from two PTMs, one of 14 Da and other of 16 Da (supplementary Fig. S6), possibly corresponding to methylation and oxidation, respectively. Furthermore, an isotopic envelope could be detected near this identified proteoform (Δm = 30 Da) with the intact mass of the −2 Da modification of HMADP (supplementary Fig. S6**,** precursor in blue).

The two putative modifications of HMADP proteoforms are close together in the protein sequence (supplementary Fig. S6). Moreover, no canonical proteoform could be identified. These suggest that the presence of methylation may affect disulfide bond formation. The HMA sequence is found in bacterial proteins conferring toxic heavy metal resistance^42^. To clarify *C. glutamicum* HMADP function, the Q8NL68 protein sequence was blasted against the UniprotKB reference proteomes plus Swiss-Prot database with default parameters in Uniprot, resulting in similarity to copper chaperone and heavy metal transport/detoxification proteins (supplementary Table 3). The transcripts for the copper-responsive two-component system, CopRS, were shown to be up-regulated in *C. glutamicum* during penicillin induction of glutamic acid production^43^. Recently, it was demonstrated that copper can induce glutamic acid production by *C. glutamicum*, however in a lower amount compared to typical treatments, such as penicillin or biotin limitation^44^. Despite this, the role of HMADP in L-glutamate production remains unclear, however it appears to be a stress response related protein, and it may therefore be regulated or regulate other proteins through PTMs associated with oxidative stress.

### Stress response related proteins

Less common Δm values were observed in a peroxiredoxin (Q8NMS6). One proteoform with a mass shift of 154 Da was identified and N-terminal acetylation was also detected (supplementary Fig. S7). Inspection of the MS/MS spectrum allowed us to identify fragments that support the 154 Da mass shift, however, the N-acetylation could not be narrowed down to a shorter sequence region, making it unclear if it was a series of methylation events or indeed an acetylation (supplementary Fig. S7). Considering only one PTM, the mass shift of 154 Da may be caused by three events: addition of glycerophosphate (UnimodAC: 419, Δm = 154.003110 Da), decanoyl (UnimodAC: 449, Δm = 154.135765 Da) or 4-oxo-2-nonenal (ONE) (UnimodAC: 721, Δm = 154.099380 Da). The ONE protein modification is a lipid peroxidation product, caused by reactive species interactions with membrane lipids, suggesting this protein may be localized near the cell membrane of *C. glutamicum*. This molecule can be added to nucleophile amino acid residues^45^. Observing the proposed region of modification, the presence of ONE at suggested site is unlikely, however, near this location are three possible modification sites, C51, K48, and C46 (supplementary Fig. S7). This peroxiredoxin was described as a peroxiredoxin Q with an important role in the oxidative stress response, and capable of reducing H_2_O_2_, t-BOOH, cumene hydroperoxide, and peroxynitrite. Interestingly, the C46 proposed to suffer the 154 Da mass shift is the catalytic cysteine of this peroxiredoxin^46^. This suggests this modification may have an important impact on its catalytic activity, and consequently in several *C. glutamicum* oxidative stress responses. Moreover, proteins involved in oxidative stress regulation are often susceptible to oxidative modifications as a process to regulate redox activity in the cell^24^. This evidence supports the 154 Da mass shift as a ONE modification in *C. glutamicum* peroxiredoxin. However, other possibilities cannot be discarded. Another peroxiredoxin proteoform with a Δm of 186 Da supports the identification of the 154 mass shift in this protein and is likely caused by a second PTM in its sequence of approximately 32 Da. In agreement, near the precursor fragmented for the identification of the 186 Da Δm proteoform, there was an isotopic envelope corresponding to the loss of around 32 Da (supplementary Fig. S7). This suggests the presence of a PTM of 32 Da in addition to the putative ONE modification. Mass differences of 32 Da are usually due to two oxidations in different methionines, but the methionines in this protein sequence are closer to the C-terminal, where several fragments were identified without this mass shift. Another possibility for this mass shift would be a dihydroxylation of cysteine (UnimodAC: 425, Δm = 31.989829 Da). There are two cysteines near the modified region (supplementary Fig. S7). Despite the pKa of free cysteine being around 8.6, the presence of positively charged residues near the cysteine decreases it by 3–4 units, supporting the oxidation of its thiol group^47^. In agreement, there is an arginine (R54) and lysine (K47) near C51. Moreover, the C51 is the resolving residue and was suggested to be very important in the catalytic process of this peroxiredoxin^46^.Two oxidations of cysteines result in the formation of sulfinic acid, which is an irreversible modification and signal for protein degradation^47^. Therefore, there is a good chance that the peroxiredoxin protein of *C. glutamicum* undergoes oxidative modifications which could regulate its activity and integrity. Furthermore, the suggested modification by a ONE peroxidation suggests a possible role of this protein in cell membrane repair.

Another thioredoxin (Q8NLG6, trxB1), described as thiol-disulfide isomerase and thioredoxins, was identified by two proteoforms, both with N-terminal cleavage of 26 amino acid residues. Interestingly, one proteoform was identified with approximately −2 Da mass shift in a region near two cysteines (supplementary Fig. S8), suggesting the formation of a disulfide bond. Another proteoform was identified with a Δm of 29.88 Da with ambiguous possibility of modifications (supplementary Fig. S8), such as two oxidations plus a disulfide bond, or methylation followed by oxidation. Moreover, both proteoforms were identified by good quality MS/MS spectra with several fragments representing the two modified forms (supplementary Fig. S8). This thioredoxin was demonstrated to be responsive to disulfide stress, regulated by the SigM sigma factor in *C. glutamicum*^48^. Thioredoxins are known to act as a repair system of oxidized cysteine residues, through its CxxC motif. Its cysteine residues undergo oxidation, forming a disulfide bond, producing the reduced form of cysteine residues in the target protein^47^. The presence of a −2 Da Δm identified near this motif of trxB1 reinforces that it is a disulfide bond. Moreover, the identification of oxidized and reduced proteoforms of a thioredoxin indicates the possibility of comparative studies in quantifying these proteoforms and estimate the redox status of the cell.

Top-down proteomic analysis of the industrial workhorse *Corynebacterium glutamicum* revealed several new putative PTMs of this bacterium related to different biological processes. More precisely, 1125 proteoforms were identified, from 273 proteins. Moreover, membrane proteins and proteins involved in translation seem to be heavily susceptible to PTMs. Proteins relevant to biotechnological and metabolic processes were identified by new proteoforms, which may imply new regulation strategies. For example, the OdhI protein is involved in amino acid production and was identified by a new proteoform suggesting a putative butyrylation or crotonaldehyde peroxidation, that may affect its inhibition of ODHC, therefore influencing glutamate production. The endoproteinase mepB protein was also identified with a new proteoform, cleavage of 93 amino acids residues of its N-terminal, suggesting an undescribed mechanism of its activation in *C. glutamicum* with possible effects on cell wall metabolism. SecG membrane protein, a subunit of protein secretion system had a putative N-terminal formylation, suggesting a degradation signaling with possible consequences in protein secretion. Another membrane protein was identified with a putative N-terminal formylation, MscL, which may influence metabolite efflux. A peroxiredoxin was identified with a putative ONE modification near its catalytic cysteine residue, suggesting a role in oxidative stress responses. Another stress response protein was identified with N-terminal cleavage, that may influence disulfide stress response. The HMADP protein, implicated in glutamate producing conditions and metal transport/detoxification, was identified by two proteoforms, one with a putative disulfide bond and another with putatives methylation and oxidation, suggesting possible effects in glutamate production and/or metal detoxification. The influence of these proteoforms in *C. glutamicum* biological processes should be validated and further investigated. For instance, a recent study used genetic engineering of *C. glutamicum* OdhI, introducing mimic amino acid residues, to investigate OdhI acetylation and succinylation influence on its interaction with ODHC^40^. Another possibility would be comparative analysis coupling quantitative top-down proteomics with metabolomics, observing differences in proteoform abundances correlated with metabolite abundance changes.

## Methods

### Strain and growth conditions

*C. glutamicum* ATCC 13032 was grown in tryptic soy agar (17 g/L pancreatic digest of casein, 3 g/L papaic digest of soybean, 5 g/L NaCl, 2.5 g/L K_2_HPO_4_, 2.5 g/L glucose monohydrate, 1.5 g/L agar) and pre-culture was performed overnight in tryptic soy broth under 170 rpm agitation at 30 °C. Afterwards, the pre-culture was used to inoculate three flasks containing CGXII media [20 g/L of (NH_4_)_2_SO_4_, 5 g/L of Urea, 40 g/L of glucose, 1 g/L of KH_2_PO_4_, 1 g/L of K_2_HPO_4_, 0.25 g/L of MgSO_4_^.^7H_2_O, 42 g/L of MOPS, 10 mg/L of CaCl_2_, 10 mg/L of FeSO_4_^.^7H_2_O, 10 mg/L of MnSO_4_^.^7H_2_O, 1 mg/L of ZnSO_4_^.^7H_2_O, 0.2 mg/L of CuSO_4_, 0.02 mg/L of NiCl_2_^.^6H_2_O, 0.2 mg/L of biotin, 0.03 mg/L of protocatechuic acid]^49^ [start optical density (OD) = 1.0]. The samples were grown for 36 h under 170 rpm agitation at 30 °C. Growth measurements were assessed by OD_600_ using a spectrophotometer CLARIOstar (BMG LABTECH, Germany).

### Sample preparation for LC–MS/MS

*C. glutamicum* cultures were harvested and centrifuged at 12,000×*g* for 10 min at 21 °C. Pellets were washed with CGXII medium minus glucose, by resuspending the pellet and centrifuging at 12,000×*g* for 10 min at 21 °C. Cells were then resuspended in lysis buffer (100 mM Tris–HCl, 2 mM DTT, SDS 4%, cOmplete™ EDTA-free Protease Inhibitor Cocktail from Roche, Basel, Switzerland), followed by physical disruption through maceration in liquid nitrogen. After maceration, the solution was centrifuged at 20,000×*g* for 15 min at 21 °C to separate insoluble particles, and the supernatant with soluble intracellular proteins was stored at −80 °C until further use. Proteins were then quantified by Qubit™ (Invitrogen). A pooled sample was prepared using equal protein quantities of each replicate and 500 µg of the pooled sample was fractionated by Gel-eluted Liquid Fraction Entrapment Electrophoresis—GELFrEE^50^, using a homemade cartridge with 12% resolving gel and 4% stacking gel. First, the sample was diluted in sample buffer 2× (Tris–HCl 0.125 M, SDS 4%, glycerol 20%, DTT 0.1 M, bromophenol blue 0.01%), then it was submitted to electrophoresis using a running buffer (Tris–HCl 0.025 M, Glycine 0.192 M, SDS 0.1%) and a constant current of 10 mA. Fractions were collected according to time (in minutes) after bromophenol blue elution (0 min). Subsequent collections were as follows: 1, 2, 3, 4, 5, 7, 9, 11, 13, 15, 20, 25, 30, 45, 60, 75, 90, and 120 min. The molecular mass range of proteins from each fraction was assessed by SDS-PAGE^51^. GELFrEE fractions were then submitted to methanol/chloroform/water precipitation for SDS removal^52^. Briefly, four volumes of methanol were added to the fractions, followed by addition of 1 volume of chloroform and 3 volumes of water, with 30 s of vortexing subsequent to the inclusion of each solution. After water addition and vortex, the solutions were submitted to centrifugation at 20,000×*g* for 10 min at 21 °C, resulting in the formation of two layers, with proteins floating between them. Then, the superior layer was removed, with care to prevent disturbing the protein pellet, and 3 volumes of methanol were added, followed by gentle mixing and centrifugation at 20,000×*g* for 10 min at 21 °C. Subsequently, the supernatant was discarded, followed by a second wash with 3 volumes of methanol as described before, and the resulting pellet was dried in a laminar flow bio-hood. After drying, pellets were resuspended in 5% acetonitrile and 0.1% formic acid and stored in −80 °C before LC–MS/MS.

### LC–MS/MS

Three technical replicates of GELFrEE fractions 0, 1, 2, 3, 4, 5, 7, 9 and 11 were submitted to top-down analysis on a nano-UHPLC Dionex Ultimate 3000 system coupled to Orbitrap Elite™ mass spectrometer (Thermo Scientific, Bremen, Germany) (LC–MS/MS). Analytical (30 cm, 75 µM I.D.) and trap (4 cm, 100 µM I.D.) columns packed with PLRPS 1000 A, 5 µM (Agilent, California, USA) were used for reverse phase separation. In the analytical column a tip of approximately 1 cm was pulled using a P-2000 instrument (Sutter, California, USA), to be used as emitter in the mass spectrometer. Both columns were kept at room temperature, controlled at approximately 22 °C. Samples were loaded using a flowrate of 3 µL/min for 10 min, under the isocratic condition of 5% Acetonitrile and 0.1% formic acid, then a gradient under flow of 0.230 µL/min was used to elute proteoforms from the column for MS analysis. The gradient was composed of solutions A (formic acid 0.1%) and B (acetonitrile, formic acid 0.1%) and was created by: 5% B (0–10 min), 20% B (10–55 min), 55% B (55–60 min), 85% B (60–80 min) and 5% B (80–90 min). Acquisitions were made during the 90 min gradient in positive mode, and the top-2 most abundant ions were fragmented by stepped high-energy collisional dissociation (HCD) applying normalized collision energy of 25, using two steps, with collision energy width of 10, resulting in two separated fragmentations of ions with normalized collision energy of 20 and 30, and analysis of all resulting fragments together in the Orbitrap. Isolation width of precursors was 25 *m/z*, using a dynamic exclusion duration of 30 s. Source-induced dissociation (SID) was used with 15 eV in MS1 and the first MS2, whilst for the second most intense ion, it was set to 35 eV. Resolutions were 240,000 and 120,000 for MS1 and MS2, respectively. The mass spectrometry proteomics data have been deposited to the ProteomeXchange Consortium via the PRIDE^53^ partner repository with the dataset identifier PXD038038.

### Proteoform identification and characterization

Thermo raw files were converted to MzML format using the MSConvert tool^54^. Identification and characterization of proteoforms were done using the TopPic suite software tool, version 1.4^55^. Briefly, spectra were deconvoluted using TopFD with default parameters followed by identifications against a *C. glutamicum* ATCC 13032 reference proteome (11-2020), obtained from UniProt (https://www.uniprot.org/). Two mass shifts between −500 Da and 500 Da were allowed in the identification and the N-terminal forms were set as “NONE” (No modifications), “NME” (N-terminal methionine excision), “NME-ACETYLATION” (N-terminal methionine excision and N-terminal acetylation), and “M-ACETYLATION” (N-terminal acetylation). A false discovery rate strategy was adopted with a decoy database, resulting in the identification of only proteoforms with FDR below 1% at the proteoform spectrum matches (PrSMs) level. Proteoform characterization and annotation were followed by visual interpretation of TopMSV^21^ files. Detected proteins’ MW evaluation and retention times were performed using VisioProt-MS^56^.

### Data analysis and functional annotation

Proteoforms identification data were analyzed in R 4.0.2 (R Core Team 2020) using the packages tidyverse^57^ and ggplot2^58^. Briefly, the number of identified mass shifts (Δm) was counted and the frequency of the most common Δm were defined based on rounded values. Then, the number of amino acid residues modified by each Δm was defined. Functional annotation and overrepresentation analysis were performed using the String Cytoscape app^59,60^, and DAVID^61^. Visual interpretation of DAVID overrepresented terms was also carried out using R computer language through the creation of a bubble plot with the tools present in ggplot2.

## Supplementary Information

Supplementary Figures.

Supplementary Table 1.

Supplementary Table 2.

Supplementary Table 3.

## Data Availability

Mass spectra raw files are available via the Proteomics IDEntifications Database Archive (PRIDE, https://www.ebi.ac.uk/pride/archive/, ID: PXD038038).
