# Supplementary material for: Revealing Corynebacterium glutamicum proteoforms through top-down proteomics
Source: Sci Rep. 2023 Feb 14;13:2602. doi: 10.1038/s41598-023-29857-6 (PMC9929327; doi:10.1038/s41598-023-29857-6)
Supplement: Supplementary file 1 — Supplementary Figures. [file 41598_2023_29857_MOESM1_ESM.docx]

Supplementary information: Revealing *Corynebacterium glutamicum* proteoforms through top-down proteomics

Reynaldo Magalhães Melo¹, Jaques Miranda Ferreira de Souza^1^, Thomas Christopher Rhys Williams², Wagner Fontes^1^, Marcelo Valle de Sousa¹, Carlos André Ornelas Ricart¹, Luis Henrique Ferreira do Vale¹*

¹Laboratory of Protein Chemistry and Biochemistry, Department of Cell Biology, Institute of Biology, University of Brasilia, Brasilia, Brazil.

²Laboratory of Plant Biochemistry, Department of Botany, Institute of Biology, University of Brasilia, Brasilia, Brazil.

*Corresponding author: [luisvale@unb.br](mailto:luisvale@unb.br)

RMM: reynaldo.melo@aluno.unb.br

JMFS: jaquess@unb.br

WF: wagnerf@unb.br

MVS: [mvsousa@unb.br](mailto:mvsousa@unb.br)

TCRW: tcrwilliams@unb.br

CAOR: [ricart@unb.br](mailto:ricart@unb.br)

LHFV: [luisvale@unb.br](mailto:luisvale@unb.br)

# Supplementary figures


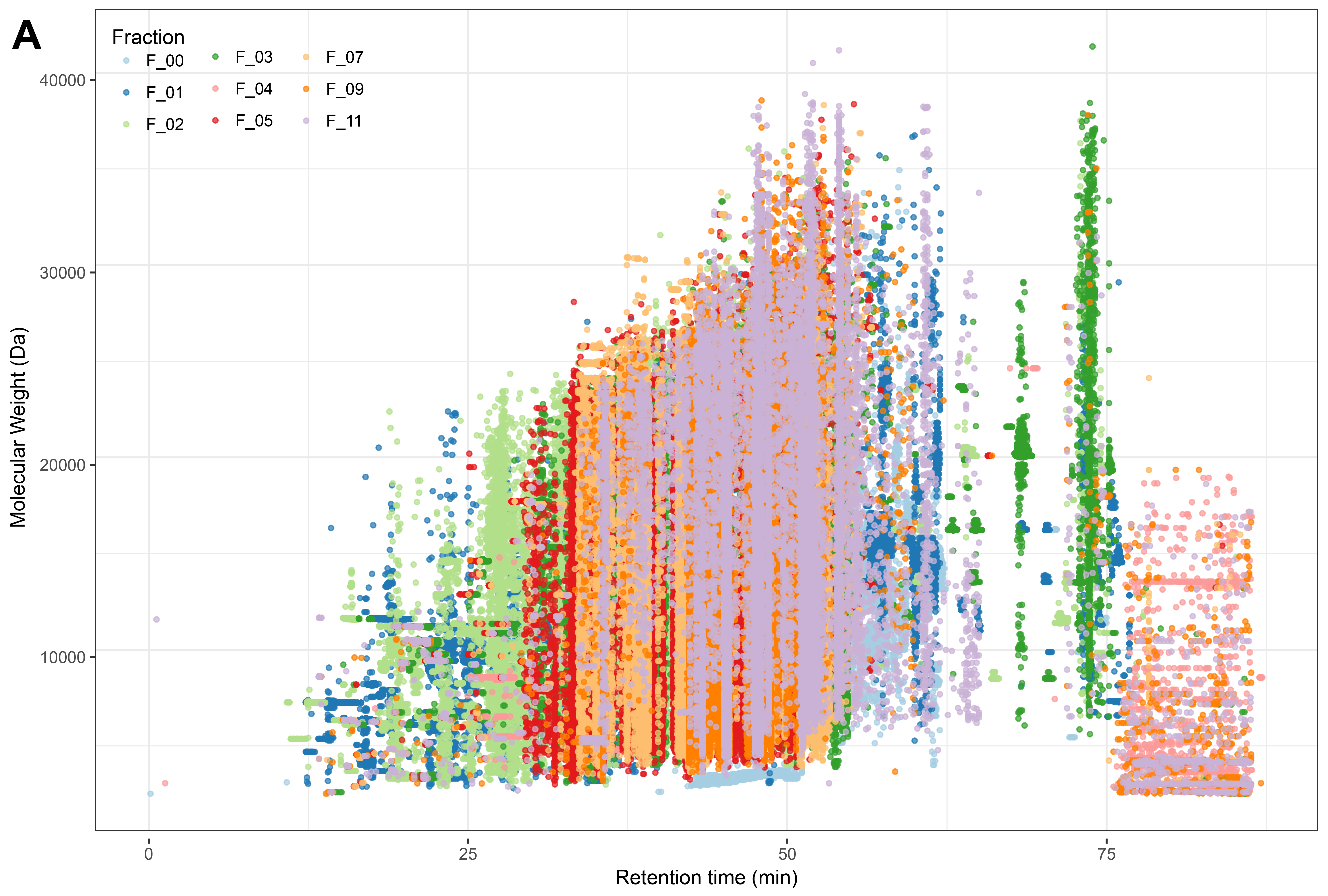


**Supplementary figure S1. Deconvoluted features mass (Da) of each fraction detected by MS1.** One replicate of each fraction was Deconvoluted by TopFD and visualized using MS-VisioProt.


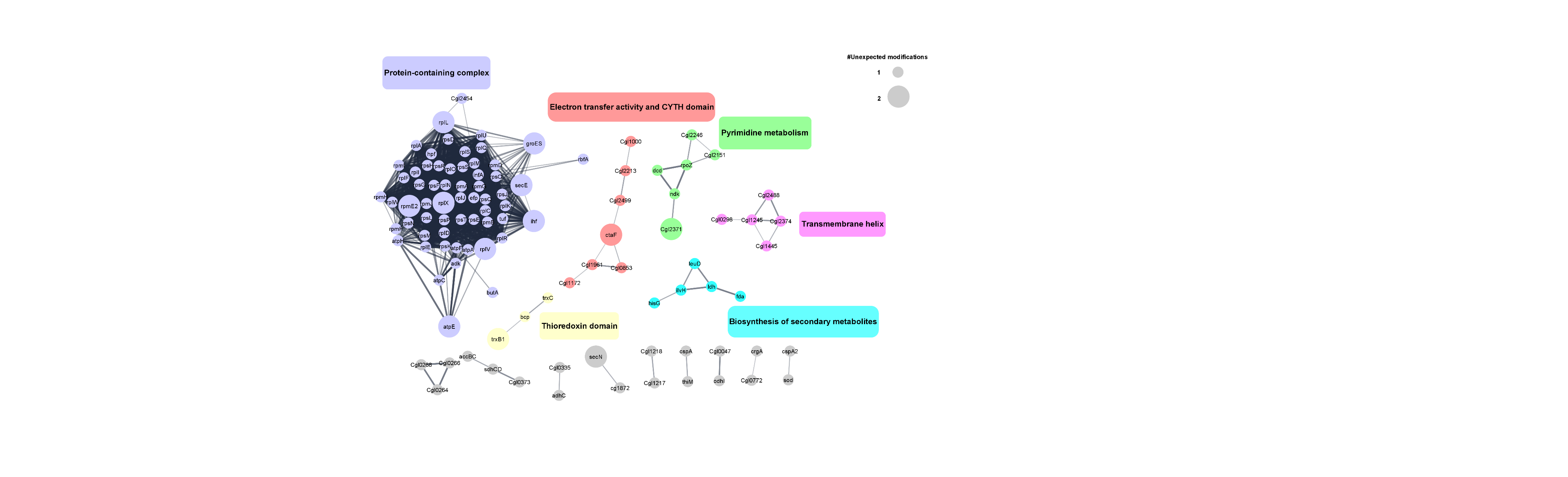


**Supplementary figure S2. Clustered proteins identified with mass shift and number of unexpected modifications identified in the same PrSM.** Proteins Uniprot Accession codes of proteoforms identified with unexpected mass shift were submitted to annotation and analysis in Cytoscape StringApp (Doncheva et al., 2019), where clustering and overrepresentation analysis of clustered groups were performed. Overrepresented terms for each cluster are represented by the colored boxes with matching colors and number of unexpected mass shifts are depicted by circle sizes.


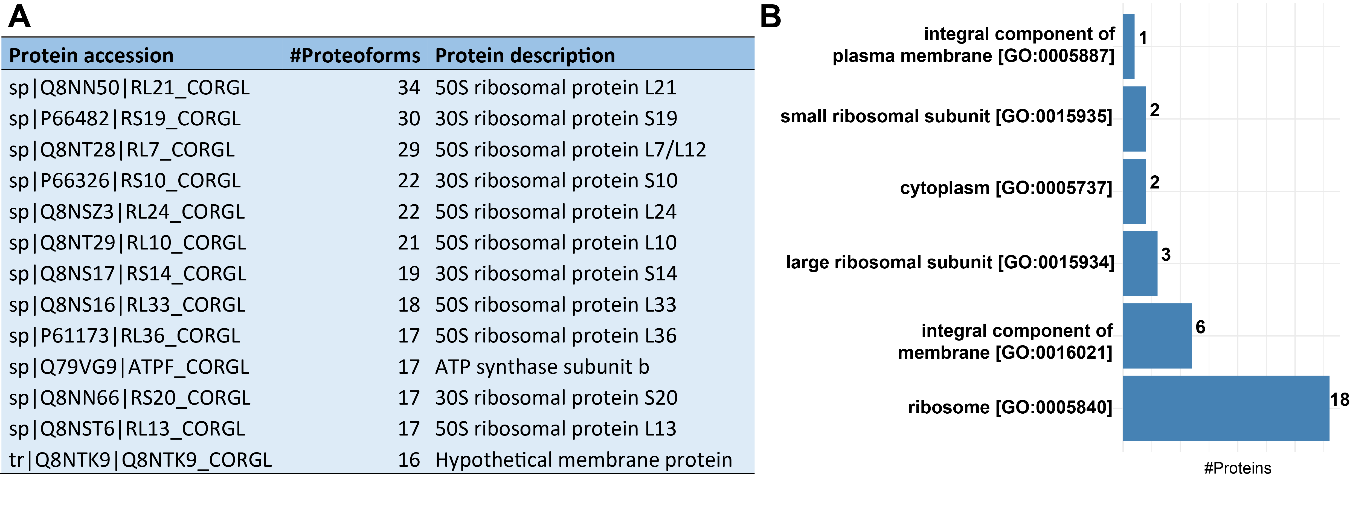


**Supplementary figure S3. Proteins with more than 15 different proteoforms cellular component annotation and description.** A) Table of Proteins that were identified with more than 15 proteoforms. B) Gene ontology, Cellular component annotation of proteins with more than 9 proteoforms.


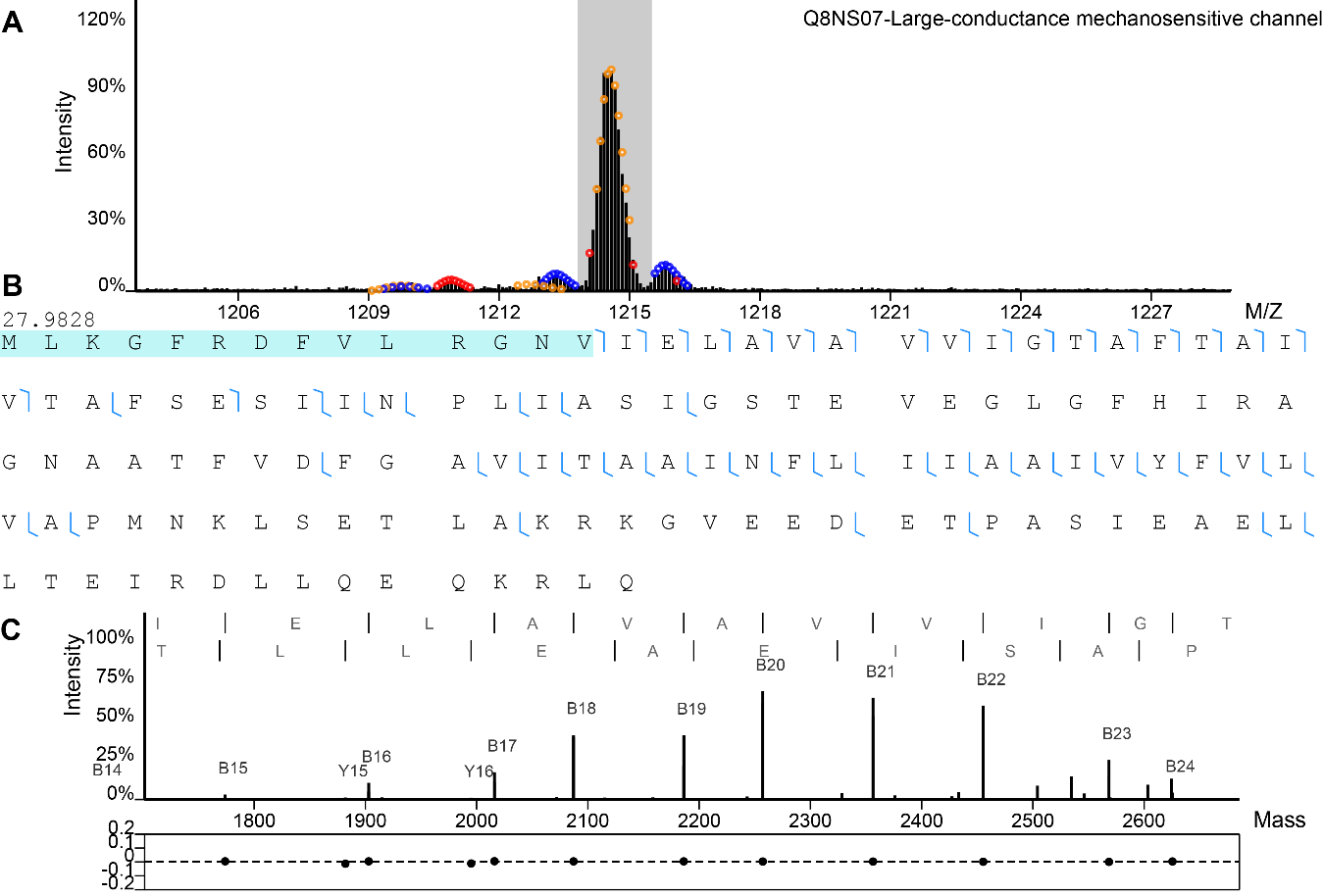


**Supplementary figure S4. PrSM of Large-conductance mechanosensitive channel (MscL, Q8NS07) with Δm of 28 Da.** Inspection of the 28 Da mass shift identified in MscL. (A) MS1 and isolation of the precursor identified as MscL. (B) Sequence of identified protein, where possible sites of the identified modification are represented by blue filling in the residues. (C) Mass representation of the MS2 spectrum (top) and mass error of matched fragments (bottom).


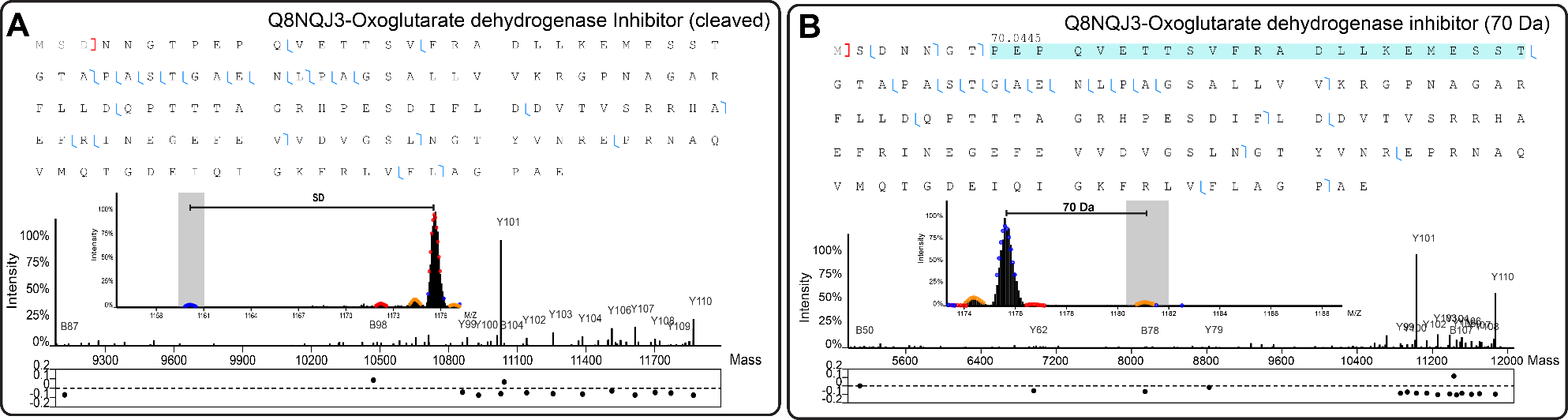


**Supplementary figure S5. Cleaved and with 70 Da Δm oxoglutarate dehydrogenase inhibitor (OdhI) PrSMs.** (A) Inspection of the cleaved OdhI proteoform. Top: Sequence of the proteoform where the identified cleavage site is represented by a red bracket. Bottom: MS1 and isolation of the precursor identified as cleaved OdhI and its mass representation of the MS2 spectrum attached to the mass error of matched fragments. (B) Assessment of OdhI proteoform with Δm of 70 Da. Top: Sequence of OdhI identified proteoform, where identified fragments are represented by blue traces in the sequence and possible sites of the Δm by blue filling. Bottom: MS1 and isolation of the precursor identified as OdhI with 70 Da Δm and its mass representation of the MS2 spectrum attached to the mass error of matched fragments.


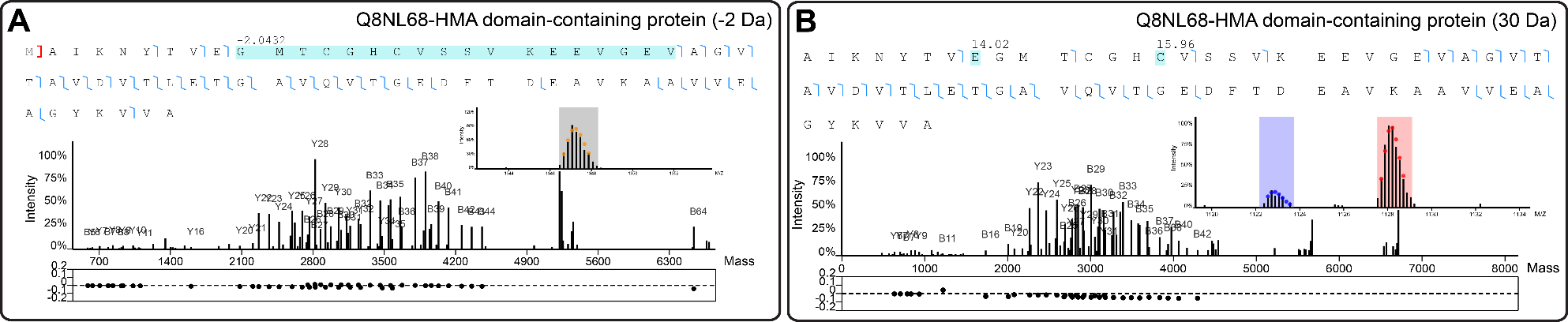


**Supplementary figure S6. Proteoforms of HMA domain-containing protein (Q8NL68, HMADP).** (A) Assessment of the -2 Da mass shift identified in HMA domain-containing protein (HMADP). Top: Sequence of identified proteoform where possible cleavage site is represented by a red bracket and Δm localization by the blue filling. Bottom: MS1, isolation of the precursor, and its mass representation of the MS2 spectrum attached to the mass error of matched fragments. (B) Assessment of HMADP proteoform with Δm of 30 Da. Top: Sequence of HMADP, where identified fragments are represented by blue traces in the sequence and possible sites of the Δm by blue filling. Bottom: MS1, isolation of the precursor (red) and an isotopic envelope with mass corresponding to the HMADP proteoform with -2 Da (blue), and its mass representation of the MS2 spectrum attached to the mass error of matched fragments.


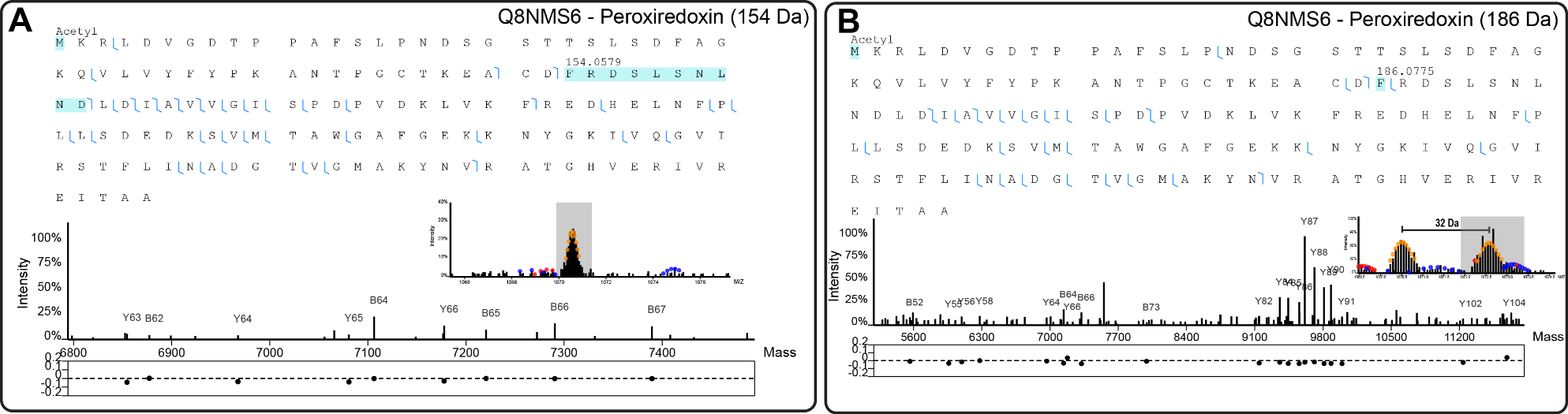


**Supplementary figure S7. PrSMs of Peroxiredoxin protein with Δm of 154 Da and 186 Da.** (A) Assessment of Peroxiredoxin PrSM with 154 Da. Top: Sequence of identified proteoform where possible mass shifts and PTMs locations are represented by blue filling. Bottom: MS1 and isolation of the precursor identified as peroxiredoxin and its mass representation of the MS2 spectrum attached to the mass error of matched fragments. (B) Assessment of peroxiredoxin PrSM with Δm of 186 Da. Top: Sequence of peroxiredoxin identified proteoform, where identified fragments are represented by blue traces in the sequence and possible sites of the Δm by blue filling. Bottom: MS1 and isolation of the precursor identified as peroxiredoxin with 186 Da Δm along with the mass difference between its nearest isotopic envelope. The mass representation of the MS2 spectrum attached to the mass error of matched fragments can be seen in the bottom of the image.


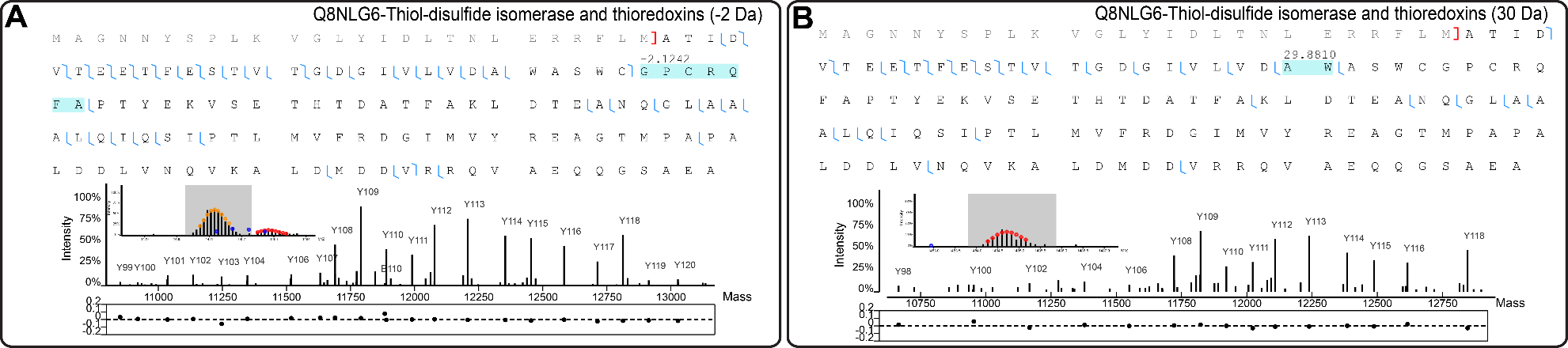


**Supplementary figure 8. PrSMs of thioredoxin protein with Δm of -2 Da and 30 Da.** (A) Assessment of thioredoxin PrSM with -2 Da. Top: Sequence of identified proteoform where possible mass shifts locations are represented by blue filling and cleavage site by red bracket. Bottom: MS1 and isolation of the precursor identified as thioredoxin and its mass representation of the MS2 spectrum attached to the mass error of matched fragments. (B) Assessment of thioredoxin PrSM with Δm of 30 Da. Top: Sequence of thioredoxin identified proteoform, where identified fragments are represented by blue traces, cleavage sites by red brackets and possible sites of the Δm by blue filling. Bottom: MS1 and isolation of the precursor identified as thioredoxin with 30 Da. The mass representation of the MS2 spectrum attached to the mass error of matched fragments can be seen in the bottom of the image

# Supplementary tables

**Supplementary table 1. List of all identified PrSMs below the 1 % FDR.** The first mass shifts of each protein spectrum match (PrSM) was extracted from the sequence information and informed in the “Mass shift (Da)” column. In PrSMs without mass shifts the term “NA” (not available) was used. The column “Putative PTM” represents post-translational modifications of the unimod database that have monoisotopic mass matching the mass shift identified in the PrSM, with a tolerance of 0.5 Da. In cases which the identified mass shift does not match the monoisotopic mass of any PTMs in unimod, the term NA was used.

**Supplementary table 2. Number of PrSMs identified with each mass shift**. The mass shift in Da was rounded to two before counting.

**Supplementary table 3. Blast results from HMADP (Q8NL68).** The sequence of HMADP was blasted against UniprotKB reference proteomes plus Swiss-Prot database using Uniprot website with default parameters.
